# Mutant p53, Stabilized by Its Interplay with HSP90, Activates a Positive Feed-Back Loop Between NRF2 and p62 that Induces Chemo-Resistance to Apigenin in Pancreatic Cancer Cells

**DOI:** 10.3390/cancers11050703

**Published:** 2019-05-22

**Authors:** Maria Saveria Gilardini Montani, Nives Cecere, Marisa Granato, Maria Anele Romeo, Luca Falcinelli, Umberto Ciciarelli, Gabriella D’Orazi, Alberto Faggioni, Mara Cirone

**Affiliations:** 1 Department of Experimental Medicine, Sapienza University of Rome, laboratory affiliated to Istituto Pasteur Italia-Fondazione Cenci Bolognetti, 00161 Rome, Italy; mariasaveria.gilardinimontani@uniroma1.it (M.S.G.M.); nives.cecere@gmail.com (N.C.); marisa.granato@uniroma1.it (M.G.); mariaanele.romeo@uniroma1.it (M.A.R.); luca.falcinelli1993@gmail.com (L.F.); alberto.faggioni@uniroma1.it (A.F.); 2 Department of Clinical Medicine, Public Health, Life and Environmental Sciences, University of L’Aquila, 67100 L’Aquila, Italy; umberto.ciciarelli@gmail.com; 3 Department of Research, Advanced Diagnostics, and Technological Innovation, Regina Elena National Cancer Institute, 00144 Rome, Italy; gdorazi@unich.it; 4 Department of Medical, Oral and Biotechnological Sciences, University “G. D’Annunzio”, 66100 Chieti, Italy

**Keywords:** apigenin, autophagy, catalase, HSP90, mutp53, NRF2, p62, pancreatic cancer, ROS, SOD

## Abstract

Pancreatic cancer is one of the most aggressive cancers whose prognosis is worsened by the poor response to the current chemotherapies. In this study, we investigated the cytotoxic effect of Apigenin, against two pancreatic cell lines, namely Panc1 and PaCa44, harboring different p53 mutations. Apigenin is a flavonoid widely distributed in nature that displays anti-inflammatory and anticancer properties against a variety of cancers. Here we observed that Apigenin exerted a stronger cytotoxic effect against Panc1 cell line in comparison to PaCa44. Searching for mechanisms responsible for such different effect, we found that the higher cytotoxicity of Apigenin correlated with induction of higher level of intracellular ROS, reduction of mutant (mut) p53 and HSP90 expression and mTORC1 inhibition. Interestingly, we found that mutp53 was stabilized by its interplay with HSP90 and activates a positive feed-back loop between NRF2 and p62, up-regulating the antioxidant response and reducing the cytotoxicity of Apigenin. These results suggest that targeting the molecules involved in the mTOR-HSP90-mutp53-p62-NRF2-antioxidant response axis could help to overcome the chemo-resistance of pancreatic cancer to Apigenin.

## 1. Introduction

Pancreatic ductal adenocarcinoma (PDAC) is the fourth leading cause of cancer death in the US mainly due to the late detection of the disease and the poor response to chemo-radiotherapies [1]. As its incidence is increasing in the recent years, the search for new and more effective treatments becomes even more urgent. Apigenin, a flavonoid widely studied for its anti-inflammatory property, has been shown to be useful in the treatment of autoimmune disorders or neurodegenerative diseases [2]. More recently, it has attracted attention also for its anticancer properties, as it has been shown to be cytotoxic against hematological [3] as well as solid cancers, in which it may induce cell death through reactive oxygen species (ROS) generation [4].

Indeed, most conventional and also non-conventional chemo- and radio-therapeutic agents can induce cancer cell death by increasing ROS [5]. The balance of ROS level depends mainly by their production by mitochondrial complexes and NADPH-oxidases (NOXs) and by the antioxidant response mediated by enzymes such as catalase, superoxide dismutase (SOD) and glutathione S-transferase (GST) which detoxify ROS and attenuate chemotherapeutic cytotoxicity [6]. Thus, sometimes, a redox resetting occurs as a protective response from tumor cells to cope with anticancer drug treatment [7]. Anti-oxidant enzyme expression is essentially regulated by the transcription nuclear factor erythroid 2 like 2 (NRF2) [8]. Activation of the NRF2-induced pathway in cancer has been shown to be critical for chemotherapeutic resistance and NRF2 is emerging as a promising target to overcome cancer chemoresistance [9]. 

Tumor suppressor p53 plays a central role in tumor prevention and response to therapies. The presence of a functional p53 pathway is incompatible with neoplastic growth, thus, p53 is the most frequently mutated gene in tumors [10]. The most prevalent missense mutations lead to a “gain of function” (GOF) that actively drives tumor progression, metastasis, and therapy resistance [11]. PDAC usually carry TP53 mutations (mutp53) [12], which promote tumor invasion and metastasis [13]. Mutp53 proteins may acquire a misfolded and partially denatured conformation forming micro- and macro-aggregates that cannot undergo degradation, with accumulation of hyperstable mutp53 proteins in tumors [14]. The most studied mechanism of mutp53 stability is the binding with the cellular chaperones heat shock proteins (HSP70, HSP90) that protect mutp53 from mouse double minute 2 homolog (MDM2)-mediated degradation. HSP90 is usually up-regulated in cancer cells to cope with the stressful conditions in which they need to survive, for this reason, HSP90 can be targeted to induce or to sensitize cancer cells to apoptosis [15]. The master regulator of the heat shock response is heat shock factor 1 (HSF1) that can also undergo interaction with mutp53 to increase HSF1-induced transcriptional program, including expression of heat shock proteins (HSPs), in a positive feed-forward loop further stabilizing mutp53 itself [16]. Of note, HSF1 and NRF2 may engage a crosstalk by sharing transcriptional targets as HSP and p62 [17] and mutp53 may interact with NRF2 differentially regulating the NRF2-mediated antioxidant response [18] making this interplay interesting for further studies as also evidenced by a mouse model where Nrf2 promotes pancreatic carcinogenesis in a background with mutant K-ras and pancreas specific mutp53 [19]. Targeting mutp53 is therefore an important anticancer strategy that has been extensively explored in the last years for degradation of mutp53 and/or reactivation of the wild-type p53 (that is inhibited by mutp53 as dominant negative effect), although with moderate success [20]. In this regard, we have demonstrated that it is possible to target mutp53 for degradation through autophagy [21,22], exploiting the effect of zinc in changing mutp53 protein conformation [23,24] or by the natural compound capsaicin [25]. As wtp53 induces autophagy while mutp53 inhibits it, likely preventing its own degradation [26], understanding the interplay between autophagy and mutp53 is important for efficient anticancer agents’ effects. Based on the above background, in this study we investigated the cytotoxic effect Apigenin against PaCa44 and Panc1 pancreatic cancer cells that display different p53 mutations, at C176S in PaCa44 and at R273H for Panc1 [27]. We also attempt to clarify the molecular interplay between mutp53, HSP90, NRF2, p62, and autophagy in regulating the response to Apigenin therapy. 

## 2. Results

### 2.1. Apigenin-Induced Cytotoxicity Correlates with ROS Increase in Panc1 and PaCa44 Pancreatic Cell Lines

**Figure 1:**
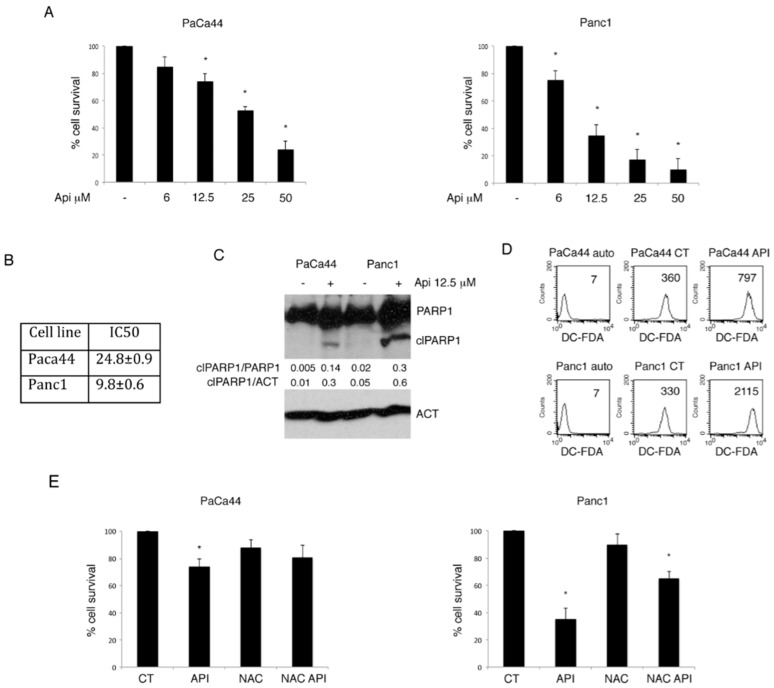
Apigenin-induced cell death is dose-dependent and correlates with reactive oxygen species (ROS) production. (**A**) Viability of PaCa44 and Panc1 treated with different doses of Apigenin (6, 12.5, 25, and 50 μM) or untreated for 24 h was evaluated by trypan blue exclusion assay. Mean plus SD of three independent experiments is reported. * *p*-value < 0.05; (**B**) IC50 (mean ± SD) of Apigenin-treated PaCa44 and Panc1 cells; (**C**) PARP1 cleavage (clPARP1) of Apigenin-treated or untreated PaCa44 and Panc1, as evaluated by western blot analysis. Actin was used as loading control. A representative experiment out of three is shown and the densitometric analysis of the ratio of clPARP1/PARP1 and clPARP1/actin is reported. (**D**) Oxidant specie production by Apigenin in PaCa44 and Panc1 evaluated by 2′,7′-dichlorofluorescein diacetate (DC-FDA) staining and assessed by Fluorescence-Activated Cell Sorting (FACS) analysis. A representative experiment out of three is shown and the mean of fluorescence intensity is indicated. (**E**) Viability of PaCa44 and Panc1 treated with Apigenin (12.5 μM), or 10 mM NAC or combination with 30 min NAC pre-treatment and Apigenin (12.5 μM) was evaluated by trypan blue exclusion assay. Mean plus SD of three independent experiments is reported. * *p*-value < 0.05; Control (CT); Apigenin (Api); cleaved Poly (ADP-ribose) polymerase (clPARP1); actin (ACT); 2′,7′-dichlorofluorescein diacetate (DC-FDA); N-acetyl-L-cysteine (NAC).

PaCa44 and Panc1 pancreatic cell lines were treated with different concentrations of Apigenin (from 6 to 50 μM) for 24 h. We found that Apigenin reduced cell survival more efficiently in Panc1 in comparison to PaCa44 cells, as indicated by the trypan-blue exclusion assay (Figure 1A). These results were confirmed by evaluating IC50 of Apigenin in both cell lines (Figure 1B). To evaluate the type of cell death, the cleavage of Poly (ADP-ribose) polymerase (PARP)1 was assessed in these cells undergoing Apigenin treatment for 24 h. The results shown in Figure 1C suggest the occurrence of an apoptotic cell death, as indicated by the appearance of the cleaved fragment of PARP1 (clPARP1) and by the increased clPARP1/PARP1 ratio, that, according to cell survival, was more evident in Panc1 cells in comparison with PaCa44 cells, at 12.5 μM of Apigenin, concentration used for all the following experiments. As Reactive Oxygen Species (ROS) increase represents a mechanism through which Apigenin may induce cancer cell death, we next evaluated ROS level in pancreatic cancer cells undergoing such treatment. We found that Apigenin induced a higher increase of ROS in Panc1 cells that correlated with the higher cytotoxic effect observed in these cells compared to PaCa44 cells (Figure 1D). The role of ROS was confirmed by the finding that pretreatment with the ROS scavenger NAC reduced the cytotoxicity of Apigenin in both pancreatic cancer cell lines (Figure 1E). All together these data suggest that the cytotoxic effect of Apigenin against the pancreatic cell lines was dependent on the increase of ROS. 

### 2.2. The Activation of NRF2-Mediated Antioxidant Response Was Responsible for the Reduced Susceptibility of PaCa44 Cells to Apigenin

**Figure 2:**
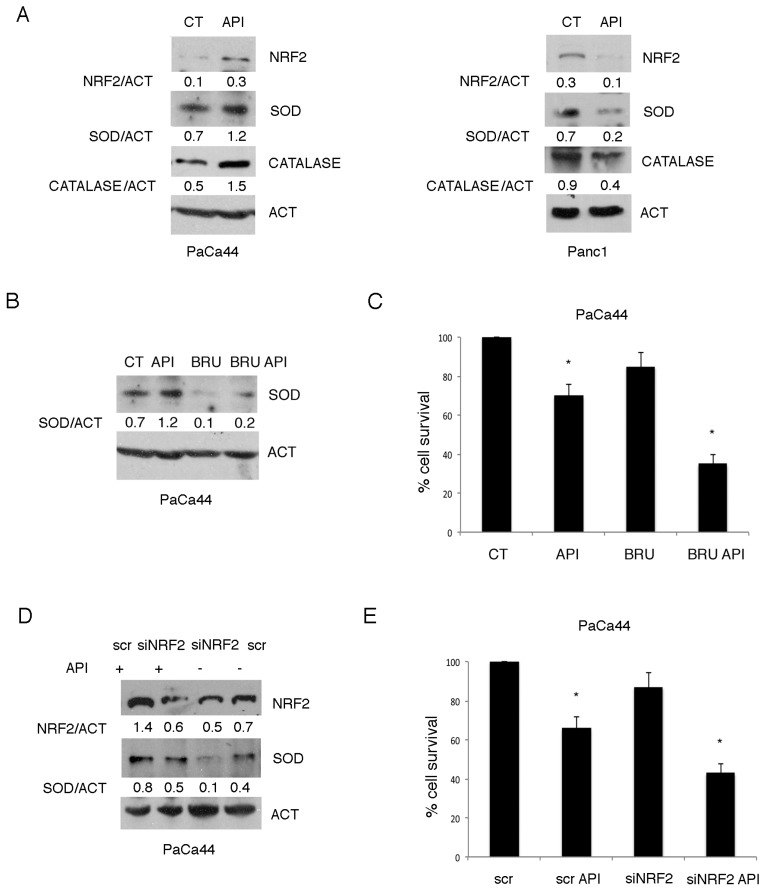
Induction of NRF2 correlates with chemoresistance to Apigenin. (**A**) NRF2, SOD, and catalase expression in Apigenin-treated or untreated PaCa44 and Panc1 cells evaluated by western blot analysis. Actin was used as loading control. A representative experiment out of three is shown and the densitometric analysis of the ratio of specific proteins/actin is reported; (**B**) SOD expression in 12.5 μM Apigenin or 30 nM Brusatol-treated or the combination of 30 nM Brusatol and 12.5 μM Apigenin and untreated PaCa44 cells evaluated by western blot analysis. Actin was used as loading control. A representative experiment out of three is shown and the densitometric analysis of the ratio of SOD/actin is reported; (**C**) Viability of untreated and 12.5 μM Apigenin or 30 nM Brusatol-treated or the combination of 30 nM Brusatol and 12.5 μM Apigenin PaCa44 cells evaluated by trypan blue exclusion assay. Mean plus SD of three independent experiments is reported. * *p*-value < 0.05; (**D**) SOD expression in NRF2-silenced or scramble PaCa44 cells treated or untreated with Apigenin was evaluated by western blot analysis. Actin was used as loading control. A representative experiment out of three is shown and the densitometric analysis of the ratio of NRF2/actin and SOD/actin is reported; (**E**) Viability of NRF2-silenced or scramble PaCa44 cells treated or untreated with Apigenin was evaluated by trypan blue exclusion assay. Mean plus SD of three independent experiments is reported. * *p*-value < 0.05. Control (CT); Apigenin (Api); nuclear factor erythroid 2 like 2 (NRF2); superoxide dismutase (SOD); actin (ACT); Brusatol (BRU); control siRNA scrambled sequence (scr); NRF2 siRNA (siNRF2).

ROS balance is regulated by their production and scavenging, mediated by the anti-oxidant enzymes including catalase and superoxide dismutase (SOD) that detoxify ROS and reduce ROS-dependent cell death following chemotherapies. Thus, we investigated whether the lower level of ROS and cytotoxicity observed in PaCa44 cells following Apigenin treatment could be due to the activation of NRF2, the most important transcription factor regulating the anti-oxidant response. We found that Apigenin increased the expression of NRF2 and its targets catalase and SOD in PaCa44 cells (Figure 2A). Conversely, the expression of NRF2, catalase and SOD was reduced by Apigenin in Panc1 cells, in agreement with the higher increase of ROS and the stronger cytotoxic effect (Figure 2A). To evaluate the role of NRF2 in up-regulating the anti-oxidant response and inducing chemoresistance, we used its inhibitor Brusatol (30 nM) before exposing PaCa44 cells to Apigenin and observed that it counteracted the up-regulation of SOD (Figure 2B) and increased cell death (Figure 2C). Similarly, knocking down NRF2 by specific siRNA reduced SOD expression and cell survival in PaCa44 cells undergoing Apigenin treatment (Figure 2D,E). All together these results suggest that the activation of NRF2 pathway was responsible for the chemoresistance to the cytotoxic effect of Apigenin in PaCa44 cells.

### 2.3. Mutp53 Expression Correlates with NRF2 Activation in Pancreatic Cancer Cells Undergoing Apigenin Treatment

**Figure 3:**
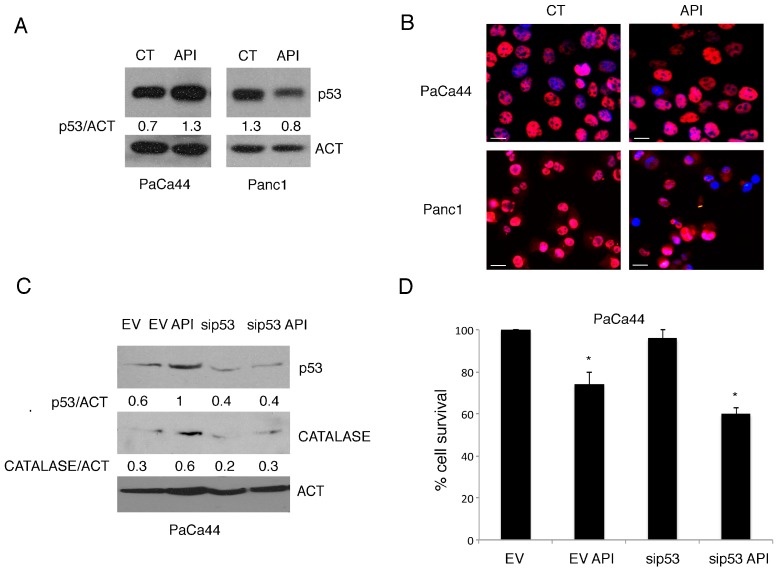
Mutant p53 expression correlates with NRF2 activation. (**A**) mutp53 expression in Apigenin-treated or untreated PaCa44 and Panc1 cells evaluated by western blot analysis. Actin was used as loading control. A representative experiment out of three is shown and the densitometric analysis of the ratio of p53/actin is reported; (**B**) mutp53 expression in Apigenin-treated or untreated PaCa44 and Panc1 cells as evaluated by IFA after 24 h of treatment; Scale bar: 10 mm. (**C**) mutp53 and catalase expression in p53- or scramble-silenced PaCa44 cells treated or untreated with Apigenin was evaluated by western blot analysis. Actin was used as loading control. A representative experiment out of three is shown and the densitometric analysis of the ratio of p53/actin and catalase/actin is reported; (**D**) Viability of p53- or scramble-silenced PaCa44 cells treated or untreated with Apigenin was evaluated by trypan blue exclusion assay. Mean plus SD of three independent experiments is reported. * *p*-value < 0.05. Control (CT); Apigenin (Api); actin (ACT); empty vector (EV); p53 siRNA (sip53).

While recent findings suggest that R273H mutp53 [28,29] may interact with NRF2 to modulate its transcriptional activity, as a novel GOF [18], no studies have so far explored the C176S mutp53/NRF2 interaction. Therefore, we next investigated the interplay between NRF2 and mutp53 in both pancreatic cancer cell lines Panc1 and PaCa44, harboring R273H and C176S mutp53, respectively. We found that Apigenin, along with the activation of NRF2, increased mut p53 expression in PaCa44 cells (Figure 3A) while down-regulated it in Panc1 cells (Figure 3A). The different modulation of mutp53 expression in Panc1 and PaCa44 cells was then confirmed by IFA that also evidenced a partial nuclear export of mutp53 in PaCa44 cells (Figure 3B). To investigate whether mutp53 could influence NRF2-mediated antioxidant response, we silenced p53 in PaCa44 cells and found that Apigenin failed to induce catalase expression (Figure 3C), suggesting that mutp53 positively regulated NRF2-mediated antioxidant response. Moreover, mutp53 silencing reduced cell survival of PaCa44 treated with Apigenin (Figure 3D) indicating that mutp53 activated the NRF2-mediated anti-oxidant response to promote chemoresistance to Apigenin. 

### 2.4. Autophagy Was Slightly Affected by Apigenin in Pancreatic Cancer Cells

**Figure 4:**
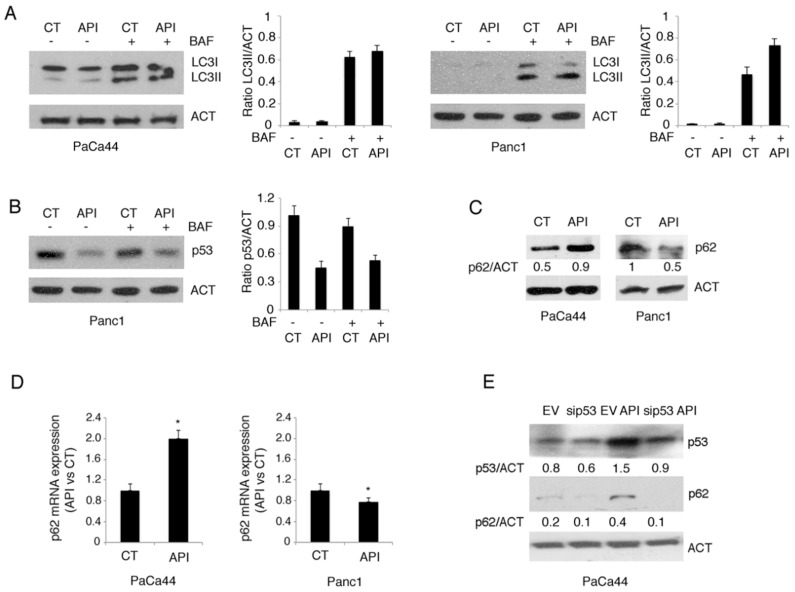
Apigenin-mediated induction of p62 is mutp53 dependent in PaCa44 cells. (**A**) LC3II expression in Apigenin-treated or untreated PaCa44 and Panc1 cells in the presence or not of 20 nM Bafilomycin (added the last 2 h) was evaluated by western blot analysis. Actin was used as loading control. A representative experiment out of three is shown and the densitometric analysis of the ratio of LC3/actin is reported; (**B**) mutp53 expression in Apigenin-treated or untreated Panc1 cells in the presence or not of 20 nM Bafilomycin (added the last 2 h) was evaluated by western blot analysis. Actin was used as loading control. A representative experiment out of three is shown and the densitometric analysis of the ratio of p53/actin is reported; (**C**) p62 expression in Apigenin-treated or untreated PaCa44 and Panc1 cells evaluated by western blot analysis. Actin was used as loading control. A representative experiment out of three is shown and the densitometric analysis of the ratio of p62/actin is reported; (**D**) p62 RNA was evaluated by qRT-PCR in Apigenin-treated or untreated PaCa44 and Panc1 cells. Target mRNA level was normalized to actin gene. Data are plotted in histograms showing standard deviation (SD). * *p*-value < 0.05. (**E**) mutp53 and p62 expression in p53-or scramble-silenced PaCa44 cells treated or untreated with Apigenin was evaluated by western blot analysis. Actin was used as loading control. A representative experiment out of three is shown and the densitometric analysis of the ratio of p53/actin and p62/actin is reported. Control (CT); Apigenin (API); bafilomycin (BAF); microtubule-associated protein light chain 3 (LC3I); lipidated microtubule-associated protein light chain 3 (LC3II); actin (ACT); empty vector (EV); p53 siRNA (sip53).

Autophagy has been reported to contribute to mutp53 degradation [21], and mutp53 has been shown to inhibit autophagy [26], therefore we then investigated the interplay between mutp53 and autophagy in Panc1 and PaCa44 cells undergoing Apigenin treatment. At this aim, LC3I/II was evaluated in these cells treated with Apigenin in the presence or in the absence of Bafilomycin A1. This is an inhibitor of vacuolar-type H^+^-ATPase that blocks the last autophagic steps and thus prevents LC3II degradation [30]. As shown in Figure 4A, Apigenin slightly affected autophagy in PaCa44 cells and minimally induced it in Panc1 cells. In the latter cells mutp53 expression, reduced by Apigenin, was slightly increased by Bafilomycin (Figure 4B) suggesting that autophagy partially contributed to the downregulation of mutp53. Next, we evaluated the expression level of p62, protein mainly degraded through autophagy, and found that its expression level was strongly reduced in Panc1 cells, while increased in PaCa44 cells (Figure 4C). These results suggest that p62 expression level was regulated independently of autophagy. Indeed, q-RT-PCR confirmed that p62 was transcriptionally up-regulated in PaCa44 and reduced in Panc1 cells following Apigenin treatment (Figure 4D). As p62 expression increased in PaCa44 cells in which also mutp53 expression was up-regulated upon treatment with Apigenin, we investigated whether mutp53 could be responsible for p62 increase. At this aim, we silenced mutp53 and found that p62 expression level decreased (Figure 4E), suggesting that mutp53 was involved in its up-regulation.

### 2.5. Mutp53 Activated NRF2 to Up-Regulate p62 that in Turn Contributes to NRF2 Activation

**Figure 5:**
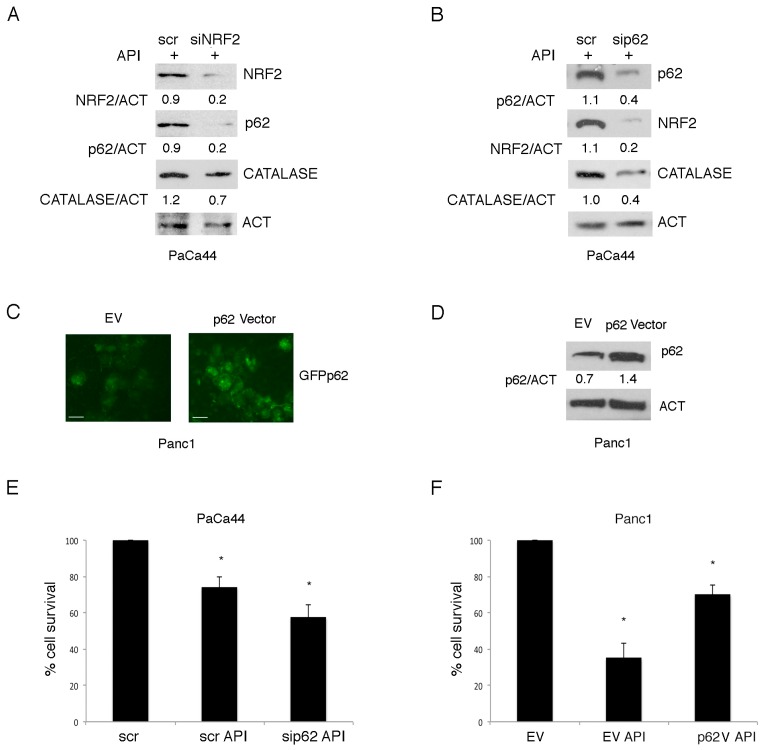
Positive feed-back loop between p62 and NRF2 mediated by mutp53. NRF2, p62, and catalase expression was evaluated by western blot analysis in (**A**) siNRF2- or scramble-silenced PaCa44 cells treated with Apigenin and (**B**) sip62- or scramble-silenced PaCa44 cells treated with Apigenin. Actin was used as loading control. A representative experiment out of three is shown and the densitometric analysis of the ratio of NRF2/actin, p62/actin and catalase/actin is reported; (**C**) Photographs of p62 positive Panc1 cells, transfected with empty vector plasmid (EV), and GFP-p62 vector plasmid observed by fluorescence microscope; Scale bar: 10 mm. (**D**) p62 expression of transfected with empty vector plasmid (EV) and GFP-p62 vector plasmid Panc1 cells evaluated in western blot analysis. Actin was used as loading control and the densitometric analysis of the ratio of p62/actin is reported. (**E**) Viability of p62- or scramble-silenced PaCa44 cells treated with Apigenin was evaluated by trypan blue exclusion assay. Mean plus SD of three independent experiments is reported. * *p*-value < 0.05; (**F**) Viability of p62- or empty-vector transfected Panc1 cells treated with Apigenin was evaluated by trypan blue exclusion assay. Mean plus SD of three independent experiments is reported. * *p*-value < 0.05. Control (CT); Apigenin (API); nuclear factor erythroid 2 like 2 (NRF2); actin (ACT); control siRNA scrambled sequence (scr); NRF2 siRNA (siNRF2); p62 siRNA (sip62); empty vector (EV); p62 vector (p62V).

The reduced susceptibility to Apigenin of PaCa44 cells correlated with the up-regulation of mutp53, the activation of NRF2 and the increase of p62. As mutp53 activates NRF2 and as p62 has been reported to be a transcriptional target of NRF2 [31], we then asked whether mutp53-mediated up-regulation of p62 could occur through the activation of NRF2 in Apigenin-treated PaCa44 cells. At this aim, we knocked down NRF2 and found that p62 expression level was reduced (Figure 5A), confirming that NRF2 was involved in p62 up-regulation induced by mutp53.

Since it has been reported by our and other’s laboratories that p62 can stabilize NRF2 to promote the antioxidant response [32,33], we hypothesized that mutp53-mediated increase of p62 could in turn activate the NRF2-mediated transcription of anti-oxidant enzymes in PaCa44 cells treated with Apigenin. To verify this hypothesis, we knocked down p62 in PaCa44 cells. As shown in Figure 5B, p62 silencing reduced the expression of catalase and NRF2 in these cells treated with Apigenin, indicating the occurrence of a positive feed-back loop between p62 and NRF2, activated by mutp53. Moreover, cell survival was reduced by p62 silencing in PaCa44 cells (Figure 5E) and, on the other hand, the introduction of the expression vector containing p62 (Figure 5C,D) partially rescued cell survival in Panc1 cells, following Apigenin treatment (Figure 5F). All together these results suggest that mutp53 activated NRF2 to promote p62 transcription and that p62 in turn contributed to NRF2-mediated activation of antioxidant response, reducing the susceptibility in PaCa44 cells to Apigenin.

### 2.6. The Expression of mutp53 Was Dependent on HSP90 Expression and Correlated with mTORC1 Activation in Pancreatic Cancer Cell Lines

**Figure 6:**
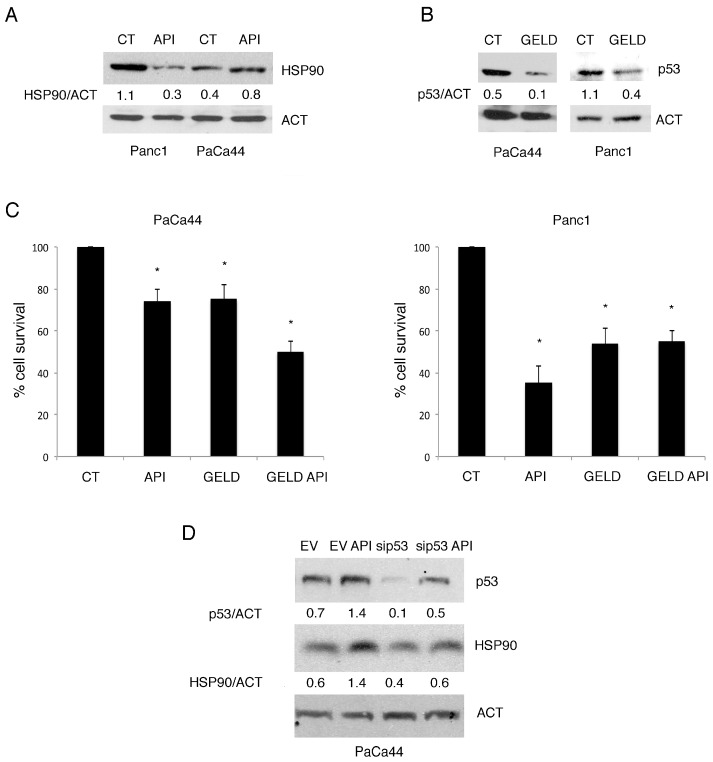
HSP90 induction by Apigenin promotes mutant p53 stability and vice versa. (**A**) HSP90 expression in Apigenin-treated or untreated Panc1 and PaCa44 cells evaluated by western blot analysis. Actin was used as loading control. A representative experiment out of three is shown and the densitometric analysis of the ratio of HSP90/actin is reported; (**B**) mutp53 expression in 100 nM Geldanamycin (GELD)-treated or untreated PaCa44 and Panc1 cells evaluated by western blot analysis. Actin was used as loading control. A representative experiment out of three is shown and the densitometric analysis of the ratio of Geldanamycin/actin is reported; (**C**) Viability of untreated or 12.5 μM Apigenin-, 100 nM Geldanamycin-treated, and the combination of 100 nM Geldanamycin and 12.5 μM Apigenin in PaCa44 and Panc1 cells was evaluated by trypan blue exclusion assay. Mean plus SD of three independent experiments is reported. * *p*-value < 0.05; (**D**) mutp53 and HSP90 expression in p53- or scramble-silenced PaCa44 cells treated or untreated with 12.5 μM Apigenin was evaluated by western blot analysis. Actin was used as loading control. A representative experiment out of three is shown and the densitometric analysis of the ratio of p53/actin and HSP90/actin is reported. Control (CT); Apigenin (API); heat shock protein 90 (HSP90); actin (ACT); geldanamycin (GELD); empty vector (EV); p53 siRNA (sip53).

Hsp90 is a molecular chaperone whose expression strongly influences mutp53 stability [34]. HSP90 has been reported to be targeted by Apigenin in multiple myeloma cells [35], thus we next investigated the relationship between mutp53 and HSP90 in PaCa44 and Panc1 cells undergoing Apigenin treatment. We found that HSP90, basally highly expressed in Panc1, was down-regulated by Apigenin while it was up-regulated PaCa44 cells (Figure 6A) in which mutp53 expression also increased. To evaluate the role of HSP90 in the stabilization of mutp53, we used the HSP90 specific inhibitor Geldanamycin and found that it reduced mutp53 expression in both PaCa44 and Panc1 cells (Figure 6B) and concomitantly reduced cell survival (Figure 6C), although the cytotoxic effect was stronger in Panc1 cells that displayed a higher constitutive HSP90 expression. These findings suggest the expression level of mutp53 was dependent on HSP90 expression. According to the previous reports showing that mutp53 may stimulate the transcription of HSPs, including HSP90, to promote its own stabilization [14], we then found that the silencing of p53 reduced the expression of HSP90 (Figure 6D). These results indicate the occurrence of an interplay between the two molecules in which HSP90 promotes mutp53 stability and mutp53 in turn promotes HSP90 expression. 

### 2.7. mTORC1 Activation Regulates Chemoresistance to Apigenin in PaCa44

**Figure 7:**
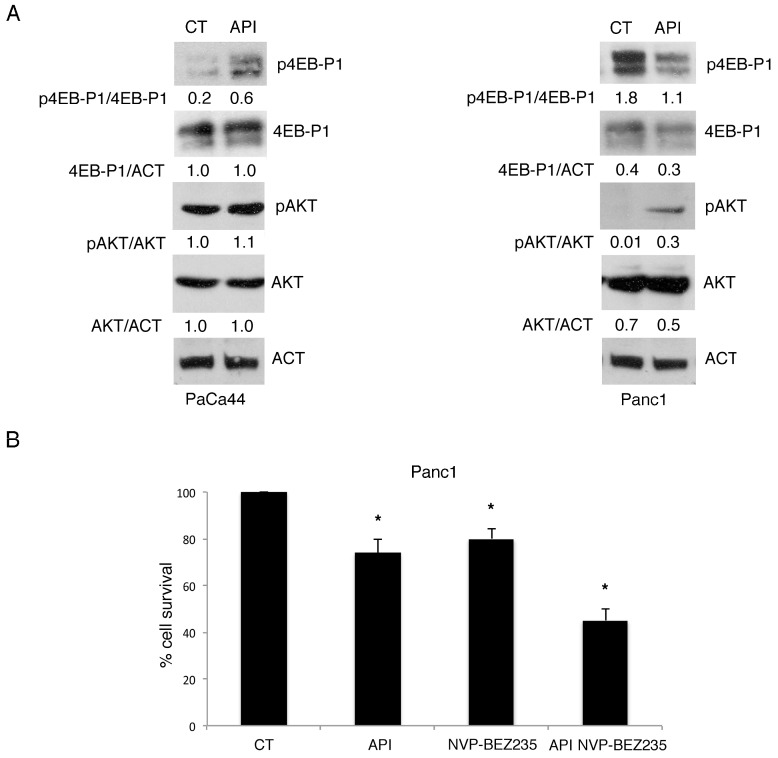
HSP90 expression is regulated by mTORC1 activation. (**A**) p4EBP1, EBP1, pAKT, and AKT expression in Apigenin-treated or untreated PaCa44 and Panc1 cells was evaluated by western blot analysis. Actin was used as loading control. A representative experiment out of three is shown and the densitometric analysis of the ratio of p53/actin is reported; (**B**) Viability of untreated or 12.5 μM Apigenin-, 100 nM dual PI3K/mTOR inhibitor NVP-BEZ235-treated, and the combination of 100 nM NVP-BEZ235 and 12.5 μM Apigenin in Panc1 cells was evaluated by trypan blue exclusion assay. Mean plus SD of three independent experiments is reported. * *p*-value < 0.05. Control (CT); Apigenin (API); eukaryotic translation initiation factor 4E (eIF4E)-binding protein 1 (4EB-P1); phospho-eukaryotic translation initiation factor 4E (eIF4E)-binding protein 1 (p4EB-P1); protein kinase B (AKT); phospho-protein kinase B (pAKT); actin (ACT).

As HSP90 expression is regulated by PI3K/AKT/mTOR axis [8], we next evaluated whether the activation of mTOR complexes (mTORC1 and mTORC2) could correlate with expression of HSP90 in Apigenin-treated pancreatic cancer cells. We found that mTORC1 target 4EBP1 phosphorylation, slightly activated in PaCa44 cells increased following Apigenin treatment (Figure 7A), in correlation with HSP90 and mutp53 up-regulation. Conversely 4EBP1 basal phosphorylation, higher in Panc1 cells, was reduced by Apigenin (Figure 7A), concomitantly with HSP90 and mutp53 down-regulation. Finally, we evaluated whether Apigenin could affect the activation of mTORC2 and found that the phosphorylation of its target AKT ser473 slightly increased in both cell lines (Figure 7A), suggesting that it did not play a role in HSP90 and mutp53 regulation by Apigenin. We then found that mTOR inhibition by the dual inhibitor of mTORC1 and mTORC2 NVP-BEZ235 counteracted the chemoresistance to Apigenin of PaCa44 cells (Figure 7B). All together these results indicate that the activation of mTORC1, correlated with the expression of HSP90 and mutp53, reduced susceptibility to Apigenin in pancreatic cancer cells. 

## 3. Discussion

Currently, chemotherapy is still one of the best therapeutic options to treat cancer, however, development of drug resistance is an important factor in anticancer therapeutic failure [36]. Pancreatic cancer is one of the most incurable cancer and gemcitabine treatment offers little potential for improving survival [37], therefore, new therapeutic options are urgently needed to in order to improve both survival and quality of life of patients with pancreatic cancer. 

**Figure 8:**
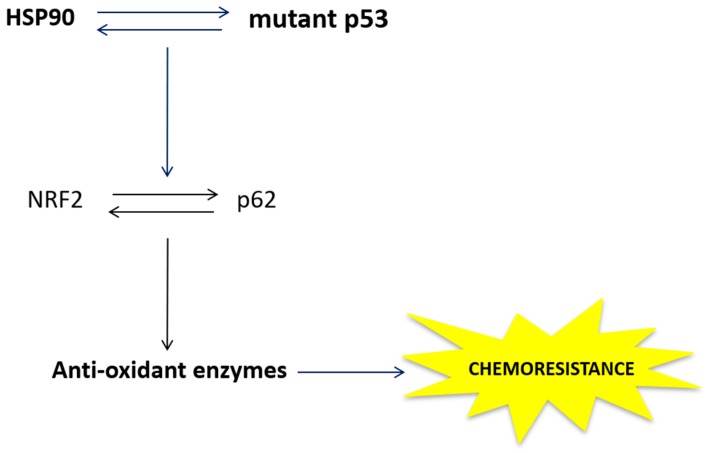
Schematic model describing how the cross-talk between mutant p53 and HSP90 and NRF2/p62 activates the antioxidant response to promote chemoresistance. Such cross-talk was promoted by Apigenin in PaCa44 cells while inhibited in Panc1 cells. Heat shock protein 90 (HSP90); nuclear factor erythroid 2 like 2 (NRF2).

The use of natural compounds that have shown remarkable anti-cancer effects without side effects have gained more and more interest in anticancer agent development [38,39]. Apigenin is a common dietary flavonoid that is abundantly present in many fruits and vegetables and was reported to suppress various human cancers in vitro and in vivo by multiple biological effects [40]. This study suggests that Apigenin may represent a valid therapeutic approach for the treatment of pancreatic cancer. It has been previously reported that Apigenin induced apoptosis in pancreatic cancer cells harboring p53 mutation by reactivating wild type (wt) p53 [41]. Differently from this study, here we could not detect the expression of wt p53-targets p21 or puma (data not shown) while we found that Apigenin cytotoxicity correlated with ROS increase. Moreover, we found that the cytotoxic effect of Apigenin was stronger in pancreatic cancer cells in which the basal mutp53 and HSP90 expression was higher and mTORC1 activated. Hot spot p53 mutations, such as that occurring at R273H, have been reported to activate mTORC1 [26] and interestingly, the activation of mTOR may promote the expression of HSP90 [8]. In agreement with these findings, we found that Panc1 cells, that displayed R273H p53 mutation, showed a basal activation of mTORC1 and a higher expression of HSP90 that rendered these cells more susceptible to Apigenin that indeed inhibited mTOR and reduced both HSP90 and mutp53 expression in these cells. On the contrary, the lower Apigenin cytotoxicity, observed in PaCa44 cells, was dependent on its-mediated increase of HSP90 and mutp53 expression occurring in these cells in which the basal activation of mTORC1 and the expression of mut p53 and HSP90 was lower. In the attempt to shed more light into the mechanisms of chemoresistance to Apigenin of PaCa44 cells, we found that interplay between mutp53 and HSP90, that reciprocally influenced their expression, led to the activation of NRF2 and the up-regulation of p62, activating a positive feed-back loop between these molecules (Figure 8). Indeed, NRF2 up-regulated the expression of antioxidant enzymes, such as catalase and SOD that maintained moderate the increase of ROS and reduced Apigenin cytotoxicity. It is known that mutp53, besides exerting a dominant negative effect on wt p53, may acquire oncogenic properties by interacting with pro-survival transcription factors, including NRF2 [18]. NRF2 is emerging as a promising target to counteract chemoresistance [42] and accordingly, we have recently found that in hyperglycemic conditions, the activation of NRF2 is responsible for the reduced response to adriamycin treatment of colon cancer cells (unpublished data). Interestingly, NRF2 may engage a cross-talk with other transcription factors such as HSF1 [17], further promoting cancer cell survival. However, it is known that mutp53 may also directly interact with HSF1 to induce HSP transcription that, among other pro-survival effects, may promote mutp53 stabilization [14]. Mutp53 activity may be influenced by its intracellular localization, as for example when in the cytoplasm, it seems to negatively regulate autophagy [43]. However, in pancreatic cancer cells, it has been reported that mutp53 inhibited autophagy independently of its localization, by leading to the activation of mTOR [26]. This is likely because autophagy may be a route through which mutp53 may be degraded [24]. In this study, we found that autophagy was slightly activated by Apigenin in Panc1 cells, along with the inhibition of mTORC1, mutp53 down-regulation and its partial nuclear export. According to this finding, autophagy slightly influenced mutp53 expression that was instead strongly dependent on HSP90 expression, a chaperone that is involved in its stabilization and whose expression may be promoted by mutp53 [14]. Therefore, we suggest that interrupting the interplay between of HSP90 and mutp53 could be a promising strategy to interfere with the downstream activation of the cross-talk between NRF2 and p62 that reduces the cytotoxic effect of chemotherapies, like Apigenin. 

## 4. Materials and Methods

### 4.1. Cell Culture and Reagents

The human pancreatic cancer cell lines Panc1 and PaCa44, were obtained respectively from the American Type Culture Collection (Rockville, MD, USA) and from Dr. M. v. Bulow (University of Mainz, Mainz, Germany). The pancreatic cell lines were grown in 5% CO_2_ saturated humidity at 37 °C and cultured as monolayers in RPMI 1640 (Corning) supplemented with penicillin/streptomycin, 2 mM L-Glutamine and 10% FBS (Euroclone, Pero (MI), Italy). Cells were always detached using Trypsin-EDTA solution (Biological Industries, Cromwell, CT, USA). Apigenin was purchased from Sigma-Aldrich (Pero (MI), Italy). 

### 4.2. Cell Viability

PaCa44 and Panc1 cells were plated in 6-well plates at a density of 2 × 10^5^ cells/well. Then, the following day, when the cells were in the exponential growth phase, cells were treated with Apigenin at different doses: 6, 12.5, 25, and 50 μM. After 24 h, a trypan blue (Euroclone, Pero (MI), Italy) exclusion assay was performed to test cell viability. Live cells were counted by light microscopy using a Neubauer hemocytometer. All the following experiments were performed with the dose of 12.5 μM Apigenin. In some experiments’ cells were pre-treated with 10 mM ROS scavenger NAC (N-acetyl-L-cysteine) for 30 min. When necessary, 20 nM Bafilomycin A1 (Baf), an inhibitor of vacuolar-H^+^-ATPase, (Santa Cruz Biotechnology Inc., Dallas, TX, USA) was added the last two hours. The NRF2 inhibitor Brusatol (Sigma-Aldrich), the HSP90 inhibitor Geldanamycin (Santa Cruz Biotechnology), and the mTOR inhibitor NVP-BEZ235 (Selleckchem, Houston, TX, USA) were used respectively at 30 nM, 100 nM, and 100 nM for 24 h. 

The concentration at which Apigenin exerts half of its maximal inhibitory effect on cell survival (IC_50_) was calculated by using GraphPad Prism 8 (Version 8.1.1, San Diego, CA, USA).

### 4.3. Western Blot Analysis

After every treatment, 1 × 10^6^ of PaCa44 and Panc1 cells were lysed, subjected to electrophoresis and transferred to nitrocellulose membranes, as previously described [44]. Membranes were blocked in PBS-0.1% Tween 20 solution containing 3% BSA, probed with specific antibodies and developed using ECL Blotting Substrate (Advansta Corporation, San Jose, CA, USA). The following antibodies were used: anti-p53, anti-sod2, anti-catalase, anti-HSP90, anti-NRF2, and anti-PARP1 (Santa Cruz Biotechnology), anti-p62 (BD Transduction Laboratories, San Jose, CA, USA) anti-LC3, anti-p-AKT and anti-AKT (Novus Biologicals, Centennial, CO, USA), anti-p-4EBP1 and anti-4EBP1 (Cell Signaling Technology, Leiden, The Netherlands), and anti-beta actin (Sigma Aldrich). Goat anti-mouse IgG-HRP and anti-rabbit IgG-HRP (Santa Cruz Biotechnology Inc) were used as secondary antibodies. 

### 4.4. Measurement of Intracellular Reactive Oxygen Species Production

To measure reactive oxygen species (ROS) production, the 2′,7′-dichlorofluorescein diacetate (DCFDA; Sigma-Aldrich D6883) 10 μM was added to cell cultures for 15 min and analyzed by FACScalibur flow cytometer (BD Transduction Laboratories), using CELLQuest Pro software (version 6.0, BD Biosciences). For each analysis 10,000 events were recorded.

### 4.5. Indirect Immunofluorescence Assay (IFA)

To detect p53, PaCa44, and Panc1 cells were grown on coverslips covered with 2% gelatin (Sigma). After 24 h, cells were treated with 12.5 μM Apigenin for additional 24 h. Then cells were washed once with PBS and fixed with 2% paraformaldehyde (Electron Microscopy Science, Hatfield, PA, USA) for 30 min and permeabilized with 0.2% Triton X-100 (Sigma Aldrich)/PBS for 5 min at RT. Then cells were incubated with a primary monoclonal antibody against p53 (Santa Cruz Biotechnology), for 1 h at RT, washed with PBS and incubated with the secondary antibody Texas Red-conjugated goat anti-rabbit IgG (Jackson ImmunoResearch Europe Ltd., Ely, UK) for 30 min. Cells were also stained with DAPI (4′,6′-diamidino-2-phenylindole) (1 μg/mL) (Sigma-Aldrich) and slides were observed by fluorescence microscope (Olympus BX53, Center Valley, PA, USA).

### 4.6. p53, p62 and NRF2 Silencing and p62 Overexpression

1.5 × 10^6^ PaCa44 cells were transfected with empty vector and sip53 plasmid for p53 knockdown or with sip62 siRNA (Santa Cruz Biotechnology, sc-29679) and siNRF2 siRNA (Santa Cruz Biotechnology, sc-37030) knockdown by electroporation using the Bio-Rad Pulse Controller at 180 Volts, according to the manufacturer’s instructions, and cultured in 24 well plates. Control siRNA-A (sc-37007 Santa Cruz Biotechnology) was used as a scrambled control. For sip62 overexpression, Panc1 was transiently transfected with empty vector (EV) or pDest-mCherry-EGFPp62 plasmid (kindly provided by Terje Johansen) [45] by electropotration using the Bio-Rad Pulse Controller, as above reported. After 24 h cells were treated with Apigenin (12.5 µM) for additional 24 h. After that period, PaCa44 cells were lysed and protein extracts were subjected to electrophoresis, as described above, while Panc1 cells were observed by fluorescence microscope (Olympus BX53, Center Valley, PA, USA). 

### 4.7. RNA Extraction

Apigenin-treated and untreated PaCa44 cells were centrifuged and washed twice in cold 1× PBS. Then, cells were lysed using 1 mL TRIzol reagent (Thermo Fisher Scientific, Waltham, MA USA) for 5 min at room temperature. Following this, 0.2 mL chloroform was added to the solution. To isolate total RNA, the mixture was centrifuged, and 0.5 mL isopropanol was added to the colorless upper aqueous phase. Extracted RNA was washed twice in 75% ethanol and then re-suspended in warmed RNAase- and DNAse-free water. To remove contaminating genomic DNA, 5 μg RNA was incubated with DNase I, according to the manufacturer’s instructions (Sigma Aldrich). RNA samples were collected and stored at −80 °C.

### 4.8. Reverse-Transcription and Quantitative PCR (qRT-PCR) 

1 μg total RNA was used to synthesize single-stranded cDNA, using SuperScript III Reverse Transcriptase Kit (Thermo Fisher Scientific, Waltham, MA, USA). A total of 2 μL/well (10 ng/sample) of template was mixed to SYBR Master (Applied Biosystems), RNAase-/DNAse-free water and primers in 20 μL volume. To perform qRT-PCR, the following conditions were used: 50 °C for 2 min, 95 °C for 2 min, 95 °C for 15 s, and 60 °C for 1 min (40 cycles). The following primers were used:

p62/SQSTM1 (FW GGAGCCAGAGAACAAGTACC; RW CTCGCTCTTTCAGTTTCATGTTC)

ACT (FW TCACCCACACTGTGCCATCCTACGA; RW CAGCGGAACCGCTCATTGCCAATGG)

Target mRNA level was normalized to housekeeping mRNA actin and analyzed, comparing to untreated sample.

### 4.9. Densitometric Analysis 

The quantification of proteins bands was performed by densitometric analysis using the Image J software (1.47 version, NIH, Bethesda, MD, USA), which was downloaded from NIH website (http://imagej.nih.gov).

### 4.10. Statistical Analysis 

Data are represented by the mean ± standard deviation (SD) of at least three independent experiments and two-tailed Student’s *t*-test was used for statistical significance of the differences between treatment groups. Difference was considered statistically significant when *p*-value was ≤ 0.05. 

## 5. Conclusions

In conclusion, this study indicates that Apigenin may induce pancreatic cell death by increasing intracellular ROS. However, we found the cytotoxic effect may vary depending on the cell lines. Searching for the mechanisms that regulate the susceptibility to Apigenin, we showed that cells with constitutive activation of mTORC1 and higher expression of HSP90 and mutp53, molecules that regulate each other’s expression, had a better response to this treatment. Indeed, Apigenin interrupted the interplay between them in these cells, down-regulating both HSP90 and mutp53 expression. Conversely, in cells displaying lower basal activation of mTORC1 and lower expression of mutp53 and HSP90, Apigenin increases their expression, leading to the activation of a cross-talk between NRF2 and p62 up-regulating the antioxidant response and preventing, to some extent, the ROS-mediated cytotoxicity. This study suggests that the inhibition of the molecules involved in the mTORC1-HSP90-mut p53-NRF2-p62 axis could be used in combination with Apigenin to overcome the chemoresistance of pancreatic cancer cells to such treatment.
